# Commentary: Awareness, use, attitudes, and implementation challenges of Bayesian dose prediction software in clinical practice: a multi-organizational survey

**DOI:** 10.3389/fphar.2026.1845742

**Published:** 2026-06-12

**Authors:** Zheng Jiao, Jun Shi

**Affiliations:** 1 Shanghai Chest Hospital, School of Medicine, Shanghai Jiao Tong University, Shanghai, China; 2 G2C Consulting, San Francisco, CA, United States

**Keywords:** Bayesian dosing software, China, clinical decision support system, implementation barriers, model-informed precision dosing, therapeutic drug monitoring

## Introduction

Alghanem et al. provide a timely assessment of the implementation landscape for Bayesian dose prediction software in clinical practice (Alghanem et al., 2026). Their survey shows that awareness among surveyed clinicians was high and attitudes are generally favorable, yet routine use among respondents remained limited, highlighting an important awareness-adoption gap in model-informed precision dosing (MIPD). The most commonly perceived barriers included licensing costs, limited institutional support, insufficient familiarity with practical clinical use, inadequate integration into clinical information systems, and the need for dedicated expertise and training. By shifting attention from technical performance to real-world implementation, the study makes a meaningful contribution to both clinical pharmacology and implementation science.

Two issues, however, merit further discussion. First, the geographic composition of the sample may limit the transferability of the reported barrier profile across health systems (Alghanem et al., 2026). Second, the survey appropriately frames Bayesian software primarily as a tool for dose optimization, but this focus may underrepresent broader clinical decision-support roles that Bayesian approaches are beginning to support (Alghanem et al., 2026; Jiao et al., 2021). These considerations do not diminish the value of the original study; rather, they help define priorities for the next phase of implementation research and software development.

## China as an underrepresented implementation pathway

Although the survey was international in scope, its respondent pool was heavily weighted toward North America, which accounted for 67.4% of participants, and no respondents from China were reported (Alghanem et al., 2026). The original study did not claim to provide a definitive global or Asia-representative estimate, and we do not interpret this distribution as a limitation of the study itself. Rather, this absence identifies an opportunity for future implementation research. China should not be considered a proxy for Asia as a whole, because Asian health systems differ substantially in reimbursement, regulation, digital infrastructure, hospital information systems, and therapeutic drug monitoring (TDM) practices. The inclusion of Chinese data would therefore not complete the Asian picture by itself, but it would add a large and informative implementation setting that is currently underrepresented.

The available Chinese evidence should also be interpreted cautiously. To our knowledge, no nationally representative survey in China has directly measured awareness, use, attitudes, and perceived barriers using the same framework as Alghanem et al. (2026). Existing publications nevertheless provide indirect but relevant implementation signals. A Chinese expert consensus indicates professional recognition of MIPD and provides implementation-oriented recommendations (Jiao et al., 2021). Reports of locally developed or hospital-integrated vancomycin decision-support systems suggest that implementation in China may not depend solely on commercial software procurement (Bai et al., 2025; Gao et al., 2018; Zhang et al., 2020; Shi et al., 2023). Hospital-based studies coupling TDM with Bayesian forecasting or population pharmacokinetic approaches further suggest that implementation often occurs through TDM-centered, pharmacy-led, or antimicrobial stewardship-linked workflows (Bai et al., 2025; Gao et al., 2018; Zhang et al., 2020; Shi et al., 2023). These data do not prove that implementation barriers in China are opposite or contradictory to those reported in North America or Europe, nor do they demonstrate higher national uptake. Instead, they are hypothesis-generating evidence that the relative weight of barriers may differ. In settings where local development or institutional deployment is feasible, licensing costs may still be relevant but may not always be dominant; interoperability with hospital information systems, local validation, regulatory classification, data governance, workflow integration, and linkage with TDM or antimicrobial stewardship services may become equally important (Jiao et al., 2021; Dibbets et al., 2025).

## Beyond conventional dose adjustment

A second issue concerns the functional scope assigned to Bayesian software. The survey by Alghanem et al. (2026) appropriately focuses on dose optimization, especially for vancomycin and aminoglycosides, because these remain the most established and widely adopted applications in routine therapeutic drug monitoring (Alghanem et al., 2026). However, the broader scope of Bayesian frameworks is also relevant from an implementation perspective: clinicians may be more likely to adopt such platforms when they help solve common real-world problems beyond target attainment alone.

One such area is the interpretation of unexpected drug concentrations and managing delayed or missed doses (Jiao et al., 2021). In long-term therapy, distinguishing pharmacokinetic variability from poor adherence is clinically important because it can alter the interpretation of TDM results, influence patient counseling, and change subsequent dosing decisions. This issue is especially relevant in chronic neurological, psychiatric, and transplant pharmacotherapy, where irregular dosing is common but often difficult to identify objectively.

**FIGURE 1:**
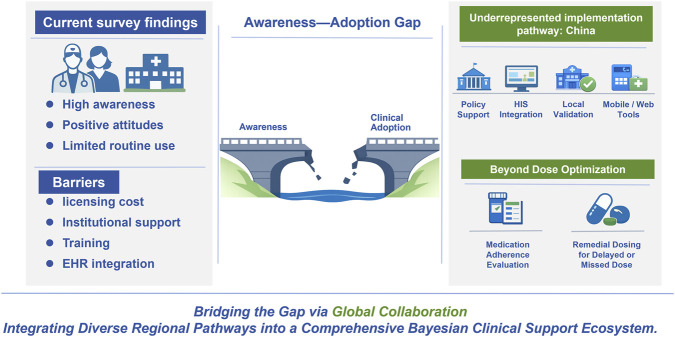
Conceptual overview of the awareness-adoption gap in Bayesian dose prediction software and the two extensions highlighted in this commentary. The left panel summarizes the main findings of Alghanem et al., including high awareness and favorable attitudes but limited clinical use, with key barriers such as licensing cost, institutional support, training, and integration challenges. The right panel highlights two underrepresented dimensions discussed here: China as a distinct implementation pathway characterized by professional guidance, hospital information system integration, and local tool development, and the expansion of Bayesian clinical decision support beyond dose optimization toward adherence assessment and missed-dose rescue. Together, these dimensions suggest priorities for future globally representative implementation surveys and software development.

Emerging methodological and applied work indicates that Bayesian approaches can support adherence assessment from sparse concentration data (Barrière et al., 2011; Liu et al., 2026) and can generate individualized remedial dosing recommendations after delayed or missed doses (Clark and Lawley, 2022; Li et al., 2023). These applications remain outside the mainstream functionality of most currently used dosing platforms and should therefore be regarded as promising but not yet widely implemented. Even so, they are clinically important because they drive a necessary evolution of Bayesian decision support from cross-sectional, single-episode dose adjustment toward longitudinal medication management. As illustrated in Figure 1, the next phase of implementation may therefore depend not only on wider uptake of current Bayesian dosing software, but also on whether such platforms evolve to address a broader range of problems encountered in everyday care.

## Discussion

These observations are intended to extend, not challenge, the contribution of the original survey. Alghanem et al. have established an important baseline for understanding how Bayesian dose prediction software is perceived and used in current practice. Our argument is not that the absence of Chinese respondents invalidates the original findings, but that it limits the ability to understand one major implementation pathway within Asia.

Future international surveys should deliberately recruit respondents from underrepresented regions, including Chinese tertiary hospitals and other heterogeneous Asian settings, and should stratify implementation models rather than treating Asia as a single homogeneous region. Specifically, future work should capture software source, procurement routes, reimbursement mechanisms, regulatory classification, local validation, hospital information system/electronic health record integration, data governance protocols, and linkage with TDM or antimicrobial stewardship services. Surveys should also ask not only who uses Bayesian software, but what they use it for: target attainment alone, or broader longitudinal decision-support tasks such as interpreting unexpected concentrations, evaluating adherence, and guiding remedial dosing. Such a design would help distinguish universal barriers to Bayesian software adoption from context-specific implementation pathways, thereby providing a more representative and actionable picture of Bayesian dose prediction software use across Asia and globally.
